# Predictive value of shear wave elastography and molecular subtypes for postoperative efficacy in patients with early breast cancer

**DOI:** 10.1080/07853890.2025.2544879

**Published:** 2025-08-18

**Authors:** Qiao-Ying Zhao, Bing Zhao, Gang Wu

**Affiliations:** a Department of Ultrasound, Henan Provincial People’s Hospital, Zhengzhou, Henan, China; b Department of Ultrasound, Zhengzhou University People’s Hospital, Zhengzhou, China

**Keywords:** Breast cancer, ultrasound elastography, molecular subtypes, disease-free survival time, overall survival time

## Abstract

**Objective:**

To investigate the predictive value of shear wave elastography and molecular subtypes for the postoperative efficacy of neoadjuvant chemotherapy (NAC) in patients with breast cancer (BC).

**Methods:**

A total of 57 patients with BC undergoing NAC were retrospectively recruited. The general and follow-up data of these patients were collected. The patients were divided into two groups: the pathological complete remission (pCR) group and the non-pCR group.

**Results:**

There were significant differences in the proportion of the elastic modulus value (*p* = 0.005), Ki-67 expression level (*p* = 0.039) and molecular subtypes (*p* < 0.001) between the two groups. Patients with a high elastic modulus value, a high Ki-67 level, as well as triple-negative and human epidermal growth factor receptor 2-positive BC were associated with poor overall survival and disease-free survival time (*p* < 0.05). Logistic regression analysis identified the elastic modulus value (odds ratio [OR]: 5.841 [3.714–6.112], *p* < 0.05), Ki-67 level (OR: 3.522 [1.865–3.897], *p* < 0.05) and molecular subtypes (OR: 4.331 [2.552–6.714], *p* < 0.05) as risk factors; logistic regression analysis also performed favourably for the prediction of pCR (area under the curve: 0.922 [0.871–0.934]) in patients with BC.

**Conclusion:**

Elastic modulus value, Ki-67 level and molecular subtypes are risk factors for pCR in patients with BC.

## 1. Introduction

Breast cancer (BC) is the leading cause of cancer death in women worldwide. Its incidence has been on the rise in recent years, and the disease is becoming increasingly prevalent among relatively young women. In 2019, a total of 304,000 women were newly diagnosed with BC in China, accounting for approximately 1/6 of all cancer incidences among women in China [1]. Therefore, more accurate prognostic indicators must be actively explored.

In recent years, increasing attention has been paid by scholars to the molecular subtypes of BC. The disease can be divided into four major molecular subtypes: luminal A BC, luminal B BC, human epidermal growth factor receptor 2 (HER2)-overexpression BC and triple-negative BC (TNBC) [2]. The frequency of luminal A and B BC, HER2-overexpression BC and TNBC were denoted as 55, 15, 15–20 and 10%, respectively [3]. Luminal BC characteristically expresses estrogen receptor (ER) with variable cell proliferation. Conversely, HER2 overexpression is the hallmark of ERBB2-overexpressing tumours, which also lack ER and progesterone receptor (PR) expression. Triple-negative BC fails to express ER, PR or HER2; instead, this type of BC expresses basal cell markers, such as cytokeratin 5/6 and/or epidermal growth factor receptor [4]. The prognosis of the four types of BC and their different molecular types is very different. Luminal A BC is the most common molecular type, with the best prognosis and the highest disease-free and overall survival (OS) rate. Although the prognosis of luminal B BC is lower than that of luminal A BC, disease-free survival (DFS) and OS rates are still higher. The prognosis of HER-2-overexpression BC and TNBC is relatively poor [5].

Recent years have seen a surge of progress in ultrasound techniques. One of these is shear wave elastography (SWE), which has been applied in clinical practice. The use of SWE can provide clinicians with the elasticity information of breast lesions [6,7]. Zhou et al. [8] conducted a study on the impact of technical factors on the evaluation of solid breast nodules via ultrasound elastography. The authors observed that the elastic data of malignant lesions obtained from elastic images of different planes varied, whereas the elastic data of benign lesions were not affected by the change of plane direction. The reason for this may be that most fibre-rich soft tissue shows varying degrees of anisotropy to the applied mechanical force [9]. Previous studies [10] have proven that SWE contributes to improving the ability to differentiate malignant breast lesions from benign breast lesions. This suggests that the significant anisotropy of BC may have a close bearing on the degree of invasion and prognostic factors. However, the SWE technique is rarely applied in the exploration of prognosis assessment and is still in the preliminary stage of research.

Multiple models have been published predicting pathological response after NAC in varying cohorts. Some baseline clinicopathological features and genotyping can predict the curative effect of NAC for breast cancer and further reflect the satisfied prognosis. However, the high costs associated with these genetic and molecular tests limit their utility in regular clinical practice. The economical and practical variables selected to establish the optimal prediction model could help select the patients who will benefit from NAC and recommend a tailored approach when choosing the initial treatment. Therefore, it is vital that more readily accessible pathologic and Shear Wave Elastography associated with NAC treatment outcomes be identified. This study aimed to evaluate the value of the SWE technique and molecular subtypes in predicting postoperative DFS time in patients with early BC.

## 2. Participants and methods

### 2.1. Participants

A total of 57 patients with early BC who underwent neoadjuvant chemotherapy (NAC) in the Oncology Department of Henan Provincial People’s Hospital (China) between April 2018 and September 2020 were retrospectively recruited as participants using the convenience sampling method.

The inclusion criteria were as follows: patients (1) pathologically diagnosed with invasive BC by core needle biopsy and with a known ER, PR, HER2 and Ki-67 status before NAC; (2) who received complete NAC with no prior treatment; and (3) with surgery performed after the completion of NAC, after which pathological complete remission (pCR) was confirmed by postoperative pathologic examination.

The exclusion criteria were as follows: patients (1) who underwent invasive examination or treatment within 1 week before peripheral blood collection; (2) with bilateral BC; (3) with evidence of active infection within the previous week; (4) with inflammatory BC; (5) suffering from any chronic autoimmune, inflammatory or haematological disease; (6) receiving less than six cycles of NAC; (7) who were pregnant or lactating; (8) with a history of prosthesis implantation; and (9) with large lesions (maximum diameter >4 cm) that could not be fully covered by the SWE colour pattern. This study was approved by the Ethics Committee of Henan Provincial People’s Hospital.

### 2.2. Criteria for molecular subtypes

Tissue samples of BA were collected via core needle biopsy for haematoxylin and eosin staining before NAC. Subsequently, the ER, PR, HER2 and Ki-67 statuses of the tumour were evaluated via immunohistochemistry (IHC) staining. The details were as follows. (1) According to the American Society of Clinical Oncology (ASCO) and the College of American Pathologists (CAP) testing guidelines [11], a tumour is considered HR-positive (ER- or PR-positive) if at least 1% of its nuclei are stained, whereas negative ER and PR are defined as HR-negative. (2) According to the ASCO/CAP HER2 testing guidelines [12], a tumour is considered HER2-positive if it is judged to be 3+ by IHC testing, or if it is determined to be 2+ and further judged to be a HER2 gene amplified by fluorescence *in situ* hybridization. (3) There is no unified method for determining the cut-off value of Ki-67. In this study, a proliferation index of ≥30% was defined as high expression, and a proliferation index of <30% was defined as low expression [13].

Criteria for molecular subtypes of BC: (1) Luminal A BC: ER- and/or PR-positive, HER2-negative; (2) luminal B BC: ER- and/or PR-positive, HER2-positive; (3) HER2-positive BC: ER- and PR-negative, HER2-positive; and (4) TNBC: ER- and PR- negative, as well as HER2-negative.

### 2.3. Shear wave elastography detection

The SuperSonic Aixplorer real-time SWE ultrasonic diagnostic instrument (SuperSonic Imagine, Aix-en-Provence, France) was used for SWE detection before NAC (SL 15-4 linear array probe, frequency: 4–15 MHz and a quantitative elastic value unit of KPa). The probe was briefly and lightly placed in the lesion area after the maximum section of the lesion was confirmed by conventional 2D ultrasound imaging. The measurement sampling frame covered the lesion as much as possible, including the entire tumour, superficial subcutaneous tissue and deep pectoral muscle structure. The patient was instructed to hold their breath after exhaling for 3 s. Stable SWE images were stored in a fixed frame after being obtained. Then, two regions of interest (Q-box) were selected for measurement, and the measurement scale was uniformly selected as 180 KPa. One region of interest focused on the hardest part of the lesion, including the immediate adjacent tissue, while a second focused on normal adipose tissue at the same depth. The maximum elastic modulus, minimum elastic modulus, mean elastic modulus and standard deviation values were quantitatively measured using two circular quantization regions of interest (Q-box). The same lesion was measured three times repeatedly, and the mean was taken as the final measurement value. All SWE measurements were conducted by two experienced radiologists who had undergone extensive training in the use of SWE technology and were blinded to the clinical data of the patients. A standardized protocol was strictly followed, including probe placement, breath-holding instructions, and selection of regions of interest. The intra-class correlation coefficient (ICC) was calculated to quantify the agreement between the two radiologists, with an ICC value of 0.85 indicating excellent inter-observer reliability. Any discrepancies in measurements were resolved through consensus reading. Additionally, the SWE instrument was calibrated daily according to the manufacturer’s guidelines, and phantom studies were conducted periodically to validate the accuracy of the measurements.

### 2.4. Neoadjuvant chemotherapy and pathological complete remission

The indications of NAC were as follows: patients (1) with stage II or III BC who underwent clinical examination for T and N staging (those who had axillary lymph nodes and were suspected of having metastatic disease on clinical examination or imaging using fine-needle aspiration or core needle biopsy); and (2) patients without contraindications to chemotherapy.

All patients underwent either 6 or 8 cycles of NAC before undergoing breast surgery, adhering to the treatment protocol outlined in the National Comprehensive Cancer Network guidelines [14]. The NAC regimens were selected based on taxane, anthracycline or a combination of both. For patients with HER2-positive BC, trastuzumab was administered, with an initial loading dose of 8 mg/kg, followed by a maintenance dose of 6 mg/kg.

At 4 weeks after the end of treatment, all patients underwent radical mastectomy. Subsequently, the final pCR was determined by standard histopathologic analysis using surgically resected specimens. The pCR was defined as the complete absence of residual invasive carcinoma in the specimen and axillary lymph nodes, regardless of the presence of residual ductal carcinoma *in situ* (ypT0/isypN0) [15].

### 2.5. Data collection

The baseline data of the participants were collected by consulting medical records, including general information and clinicopathological data. General information included age and body mass index (BMI). The clinicopathological data mainly comprised tumour location, tumour size, pathological classification, clinical stage, Ki-67 level and immunohistochemical results (ER, PR and HER2).

The follow-up outcomes included recurrence or metastasis and death. The follow-up time was calculated from the first day after surgery, and the DFS time was calculated from the first day after surgery to the first recurrence or metastasis. The total survival time was calculated from the first day after surgery to death or the final follow-up. Loss to follow-up was calculated as censored from the date of loss to follow-up.

Recurrence or metastasis refers to local recurrence or metastasis of distant organs after treatment of the primary focus as confirmed by imaging or pathological biopsy. The main prognostic indicators observed in this study were the OS and DFS rates within 27 months.

### 2.6. Statistical analysis

All data in this study were statistically processed using the SPSS 26.0 software (IBM, Armonk, NY, USA). Measurement data satisfying normality were expressed by (x ± s), the independent samples *t*-test was utilized for between-group comparison, and analysis of variance was employed for multiple-group comparison. Count data were expressed as a frequency (*n*) or rate (%) and compared using the chi-square (*χ^2^*) test. Logistic regression was used to investigate the factors affecting DFS time. The results are presented using traditional significance levels (i.e. uncorrected for multiple comparisons); selected significance levels were corrected for multiple comparisons by the Benjamini–Hochberg (BH) procedure. Furthermore, the receiver operating characteristic curve was utilized to analyze the predictive value of various factors on the postoperative DFS time in patients with BC. The Kaplan–Meier method was used to generate the survival curve, and the log-rank test was used for comparisons between the two groups. The test level was set at *α* = 0.05.

## 3. Results

### 3.1. Comparison of clinical data of patients with different efficacies

A comparison of the clinical data of patients with different efficacies showed 31 patients in the pCR group, with a mean age of 43.56 ± 5.32 years. The non-pCR group included 27 patients, with a mean age of 41.48 ± 6.32 years. No statistically significant differences were observed in terms of age, BMI, tumour diameter, tumour location, clinical stage and case classification between the two groups (all *p* > 0.05). However, there were statistically significant differences in the elastic modulus value (63.37 ± 12.23 vs. 69.04 ± 14.54, *t* = 8.063, *p* = 0.005), the high Ki-67 expression level (32.26 vs. 59.26%, *χ^2^* = 4.254, *p* = 0.039) and the proportion of molecular subtypes (*χ^2^* = 31.876, *p* < 0.001) between the two groups. See Table 1 for details.

**Table 1.** Comparison of clinical data of patients with different efficacy.

Clinical data	pCR group (*n* = 31)	Non-pCR group (*n* = 27)	*χ^2^*/*t* Value	*p* Value
Age (years old, x ± s)	43.56 ± 5.32	41.48 ± 6.32	0.998	0.612
BMI (kg/m^2^, x ± s)	21.12 ± 1.85	20.76 ± 1.73	0.533	0.811
Tumor diameter (cm, x ± s)	3.72 ± 1.03	3.58 ± 1.12	0.672	0.784
Tumor location (left/right)	14/17	14/13	0.259	0.611
Molecular subtypes			31.876	<0.001
Luminal A	12	4		
Luminal B	9	3		
Her-2 positive	6	11		
Triple negative	4	9		
Elastic modulus value (KPa, x ± s)	63.37 ± 12.23	69.04 ± 14.54	8.063	0.005
Clinical stage			1.072	0.300
II	13	15		
III	18	12		
Ki-67 expression level (high/low)	10/21	16/11	4.254	0.039
Pathological classification			0.624	0.430
Invasive ductal carcinoma	14	15		
Other invasive cancers	17	12		

*Note*: pCR: pathological complete remission; BMI: body mass index.

### 3.2. Logistic regression analysis of suspicious factors affecting pathological complete remission

Regression analysis showed that the elastic modulus value (odds ratio [OR]: 5.841, 95% confidence interval [CI]: 3.714–6.112, *χ^2^* = 9.143, *p* < 0.05), Ki-67 expression level (OR: 3.522, 95% CI: 1.865–3.897, *χ^2^* = 7.744, *p* < 0.05) and molecular subtypes (OR: 4.331, 95% CI: 2.552–6.714, *χ^2^* = 8.556, *p* < 0.05) were risk factors for a low pCR rate in patients with BC. See Table 2 for details. The BH-corrected significance levels showed significant effects on the elastic modulus value, Ki-67 expression level and molecular subtypes (at a false discovery rate of 0.05) for pCR.

**Table 2.** Logistic regression analysis of suspicious factors affecting pCR.

Variable	*β*	S.E	Wald *χ^2^*	OR	95%CI	*p* Value
Molecular subtypes	1.532	0.354	8.556	4.331	2.552 ∼ 6.714	<0.05
Elastic modulus value	1.678	0.443	9.143	5.841	3.714 ∼ 6.112	<0.05
Ki-67 expression level	1.895	0.421	7.744	3.522	1.865 ∼ 3.897	<0.05

*Note*: pCR: pathological complete remission.

### 3.3. Predictive value of the elastic modulus value, Ki-67 expression level and molecular subtypes for pathological complete remission

**Figure 1.**
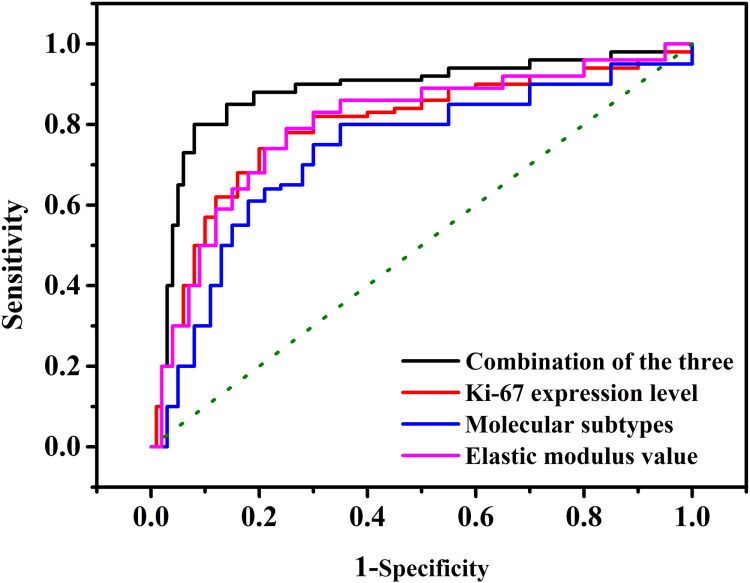
The ROC curve for regression model. The curve shows the area under the curve (AUC) of molecular subtypes for predicting the postoperative pCR of patients with BC. Sensitivity: it reflects the model’s ability to identify positive classes. Specificity: it refers to the ability of the model to correctly identify negative class samples.

The elastic modulus value, Ki-67 expression level and molecular subtypes all had a degree of predictive value for the postoperative pCR of patients with early BC (*p* < 0.05). The area under the curve (AUC) of molecular subtypes for predicting the postoperative pCR of patients with BC was 0.731 (95% CI: 0.687, 0.821), with a sensitivity of 76.34% and a specificity of 78.34 (Table 3, Figure 1). The AUC of the elastic modulus value for predicting such postoperative pCR was 0.764 (95% CI: 0.711, 0.824), with a sensitivity of 78.64%, a specificity of 80.10% and an optimal cutoff value of 66.50 kPa. In addition, the AUC of the Ki-67 expression level in predicting such postoperative pCR was 0.762 (95% CI: 0.693, 0.822), with a sensitivity of 75.43% and a specificity of 76.34% (Table 3, Figure 1). The combination of these three factors had the highest value in terms of predicting postoperative pCR in patients with early BC, with an AUC of 0.922 (95% CI: 0.871, 0.934), a sensitivity of 88.41% and a specificity of 87.42% (Table 3, Figure 1).

**Table 3.** Predictive value of molecular subtypes, elastic modulus value and Ki-67 expression level for pCR.

Variable	AUC	95%CI	Cutoff value	Sensitivity (%)	Specificity (%)
Molecular subtypes	0.731	(0.687, 0.821)	–	76.34	78.34
Elastic modulus value	0.764	(0.711, 0.824)	66.50	78.64	80.10
Ki-67 expression level	0.762	(0.693, 0.822)	–	75.43	76.34
Combination of the three	0.922	(0.871, 0.934)	–	88.41	87.42

*Note*: pCR: pathological complete remission.

### 3.4. Relationship between factors affecting pathological complete remission and outcomes

**Figure 2.**
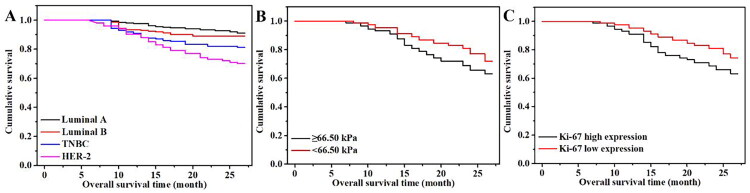
Relationship between molecular subtypes, elastic modulus value, Ki-67 expression level and OS of the two groups. (A) Overall survival time of patients of varying molecular subtypes; (B) overall survival time of patients with different elastic modulus values; (C) overall survival time of patients with different Ki-67 expression levels. Overall survival time: Time from start of patient treatment until patient death or last follow-up.

A total of 58 patients were followed up in this study, and no cases were lost. The follow-up lasted from 5 to 27 months, with a median follow-up time of 15.3 ± 5.6 months. In terms of OS, there was a statistically significant difference in the OS time of patients reflecting varying molecular subtypes within 27 months (91.10 vs. 89.10% vs. 81.20 vs. 70.20%, *p* < 0.05) (Figure 2A). Further pairwise comparison of the OS rates of the 4 molecular subtypes showed that the order from largest to smallest was as follows: luminal A BC = luminal B BC > TNBC > HER2-positive BC. The OS rate of the high elastic modulus group was lower than that of the low elastic modulus group (63.20 vs. 72.10%, *p* < 0.05) (Figure 2B). The OS rate of the high Ki-67 expression level group was lower than that of the low Ki-67 expression level group (63.21 vs. 74.30%, *p* < 0.05) (Figure 2C). The BH-corrected significance levels showed significant effects on the molecular subtypes, elastic modulus value and Ki-67 expression level (at a false discovery rate of 0.05) for OS.

**Figure 3.**
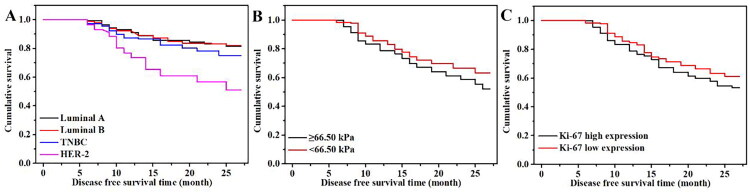
Relationship between molecular subtypes, elastic modulus value, Ki-67 expression level and DFS of the two groups. (A) Disease-free survival time of patients of varying molecular subtypes; (B) disease-free survival time of patients with different elastic modulus values; (C) disease-free survival time of patients with different Ki-67 expression levels. Disease-free survival time: Time from the initial time point when the patient receives the treatment to the first occurrence of disease recurrence, progression or metastasis, or to the last follow-up.

In terms of DFS, there was a statistically significant difference in the DFS time of patients with varying molecular subtypes within 27 months (81.50 vs. 82.21% vs. 75.20 vs. 51.11%, *p* < 0.05) (Figure 3A). Further pairwise comparison of the DFS rates of the 4 molecular subtypes showed that the order from largest to smallest was as follows: luminal A BC = luminal B BC > TNBC > HER2-positive BC. Moreover, the DFS rate of the high elastic modulus group was lower than that of the low elastic modulus group (52.11 vs. 63.30%, *p* < 0.05) (Figure 3B), and that of the high Ki-67 expression level group was lower compared with the low Ki-67 expression level group (53.41 vs. 61.20%, *p* < 0.05) (Figure 3C). The BH-corrected significance levels showed significant effects on the molecular subtypes, elastic modulus value and Ki-67 expression level (at a false discovery rate of 0.05) for DFS.

### 3.5. Multivariable Cox regression analysis for survival outcome

To further assess the independent prognostic value of the elastic modulus value, Ki-67 expression level, and molecular subtypes on OS and DFS, we performed multivariable Cox regression analyses. The elastic modulus value was an independent prognostic factor for DFS (HR: 1.025, 95% CI: 1.007–1.043, *p* = 0.005). A higher elastic modulus value was associated with a higher risk of recurrence or metastasis. A higher Ki-67 expression level was associated with worse DFS (HR: 1.038, 95% CI: 1.015–1.061, *p* = 0.001). The molecular subtypes showed significant differences in DFS. Compared to Luminal A subtype (reference group), HER2-positive subtype had a higher risk of recurrence or metastasis (HR: 2.31, 95% CI: 1.21–4.42, *p* = 0.011), and TNBC subtype also had a higher risk (HR: 2.05, 95% CI: 1.08–3.89, *p* = 0.029). Luminal B subtype did not show a significant difference in DFS compared to Luminal A subtype (HR: 1.32, 95% CI: 0.71–2.45, *p* = 0.382). The multivariable Cox regression analyses for OS and DFS confirmed that the elastic modulus value and Ki-67 expression level are independent prognostic factors for both overall survival and disease-free survival in patients with early breast cancer. The molecular subtypes, particularly HER2-positive and TNBC, also showed significant independent prognostic value. These findings further support the importance of integrating shear wave elastography and molecular subtypes in the prognostic assessment of early breast cancer patients.

## 4. Discussion

This study showed that the elastic modulus value, Ki-67 expression level and molecular subtypes all had a degree of predictive value for the postoperative pCR of patients with early BC. In addition, we also found that the DFS rate of the high elastic modulus group was lower than in the low elastic modulus group, and that of the high Ki-67 expression level group was lower compared with the low Ki-67 expression level group. This study validated the value of SWE techniques and molecular subtypes in predicting postoperative efficacy in patients with early BC.

Breast cancer is a highly heterogeneous malignant tumour. Domestic and foreign scholars have increasingly conducted studies on the prognosis and long-term survival of patients with BC of varying molecular subtypes. Onitilo et al. [16] showed that the 5-year OS rate of patients with BC of varying molecular subtypes was 90.3% for luminal A BC, 88.7% for luminal B BC, 79.0% for TNBC and 78.8% for HER2-positive BC, and the 5-year DFS rate was 86.8% for luminal A BC, 83.2% for luminal B BC, 73.5% for TNBC and 66.0% for HER2-positive BC. It has also been reported in the literature [17] that the median survival time of luminal B BC was the longest (77.6 months), while that of TNBC was the shortest (63.3 months) among the subtypes. Although the research results are not exactly the same, most scholars (both at home and abroad) agree that the prognosis of patients with luminal BC appears to be the best among the subtypes and that the prognoses of patients with TNBC and HER2-positive BC are poor. In this study, the OS rate of luminal A BC was 91.10%, and the DFS rate was 81.50%. The OS and DS rates of luminal B BC were 89.10 and 82.21%, respectively; those of HER2-positive BC were 70.20 and 51.11%, respectively, and those of TNCB were 81.20 and 75.20%, respectively. The results showed that the prognosis of luminal BC was better than that of both TNBC and HER2-positive BC.

Shear wave elastography can be used to measure tumour hardness, with a higher average hardness of tumour tissue indicating greater malignancy. Previous studies indicate that the decrease of tumour hardness by ultrasound is a good predictor of pCR detection after NAC. Evans et al. [18] confirmed that the changes in tumour hardness after 3 cycles of NAC and those monitored before chemotherapy were closely related to pCR after 6 cycles of NAC. Jing et al. [19] revealed that a change in Young’s modulus 2 weeks after NAC could predict the response of NAC, with predicted sensitivity and specificity of 73 and 86%, respectively. As shown in this study, the OS rate of the high elastic modulus group was 63.20%, and the DFS rate was 52.11% by the end of the follow-up. The OS and DFS rates of the low elastic modulus group were 72.10 and 63.30%, respectively. The results revealed that the prognosis of patients in the high elastic modulus group was worse than that of those in the low elastic modulus group, which was consistent with most studies at home and abroad.

Many studies have revealed a correlation between a high Ki-67 expression rate and various human tumours, such as BC, cervical cancer, non-Hodgkin’s disease and glioma [20]. In a study conducted by Liu Xinjie et al. [21] the IHC technique was used to measure the expression of Ki-67, ER and PR in 31 patients with locally advanced BC before and after NAC with taxanes and anthracyclines. The results showed that the expression changes of Ki-67, ER and PR were not associated with NAC. Faneyte et al. [22] found that the expression rate of Ki-67 in patients with BC decreased significantly after NAC, with a statistically significant difference. Additional research [23] also found that the expression of Ki-67 in patients with BC decreased significantly after NAC. The present study showed that the prognosis of the high Ki-67 expression level group was better compared with the low Ki-67 expression level group. The predictive value evaluation of pCR also showed that the elastic modulus value, Ki-67 expression level and molecular subtypes all had a degree of predictive value for postoperative pCR in patients with early BC (*p* < 0.05), and the combination of the three had the highest predictive value, with an AUC of 0.922 (95% CI: 0.871, 0.934), a sensitivity of 88.41% and a specificity of 87.42%.

The present study still has some limitations. First, no unified standard for the classification of BC molecular subtypes or the method of defining the molecular subtypes of tumours was proposed. Second, the results of the study are not fully in line with studies by domestic and foreign scholars; this may have resulted from the different molecular subtype standards and diverse participants. This study recruited patients with BC from a Grade-A tertiary hospital, which may have caused differences from previous studies in terms of pathological types and disease severity. In addition, our study is the relatively short follow-up duration, with a median follow-up time of 15.3 months. This limited follow-up period may not be sufficient to fully assess the long-term DFS or OS outcomes in early breast cancer patients. Longer-term follow-up is essential to provide a more comprehensive understanding of the prognostic impact of the elastic modulus value, Ki-67 expression level, and molecular subtypes on postoperative outcomes. Future studies should aim for extended follow-up periods to better evaluate the long-term survival outcomes and the sustained predictive value of these factors. Finally, this study is a single-centre retrospective study with a small sample size. Therefore, future studies on the postoperative survival status of patients with different molecular subtypes of BC should first develop a unified and effective molecular subtype method, followed by the completion of a large-sample, multi-centre study.

In summary, the elastic modulus value, Ki-67 expression level and molecular subtypes all showed a degree of predictive value for postoperative pCR in patients with early BC and negatively affected the postoperative OS and DFS times in these patients. Patients with BC who had a high elastic modulus value, high Ki-67 expression level and TNBC and HER2-positive BC had shorter OS and DFS times.

## Data Availability

The datasets used and analyzed during the current study are available from the corresponding author on reasonable request.
